# Periostin is overexpressed, correlated with fibrosis and differs among grades of cardiomyocyte hypertrophy in myectomy tissue of patients with hypertrophic cardiomyopathy

**DOI:** 10.1371/journal.pone.0293427

**Published:** 2023-11-08

**Authors:** Nikolaos S. Ioakeimidis, Antonios Pitsis, Thomas Zegkos, Dimitrios Ntelios, Timotheos Kelpis, Theodora Papamitsou, Despoina Parcharidou, Thomas Gossios, Georgios Efthimiadis, Soultana Meditskou

**Affiliations:** 1 Laboratory of Histology and Embryology, Department of Medicine, School of Life Sciences, Aristotle University of Thessaloniki, Thessaloniki, Greece; 2 Department of Cardiac Surgery, European Interbalkan Medical Center, Thessaloniki, Greece; 3 First Department of Cardiology, AHEPA University Hospital of Thessaloniki, Thessaloniki, Greece; University of Rochester Medical Center, UNITED STATES

## Abstract

Periostin, a secreted matricellular protein, has been implicated in cardiac extracellular matrix remodeling and fibrosis. Evidence suggest that periostin stimulates cardiomyocyte hypertrophy. The current study aims to investigate the extent of periostin expression in patients with advanced Hypertrophic Cardiomyopathy (HCM) and its correlation with fibrosis and hallmark histopathological features of the disease. Interventricular septal tissue from thirty-nine HCM patients who underwent myectomy and five controls who died from non-cardiac causes was obtained. Staining with Masson’s Trichrome and immunohistochemistry were used to localize fibrosis and periostin respectively. The extent of fibrosis and the expression of periostin were defined as the stained percentage of total tissue area using digital pathology software. Periostin expression was higher in HCM patients compared to controls (p<0.0001), positively correlated with the extent of fibrosis (r = 0.82, p<0.001), positively correlated with maximal interventricular septal thickness (Rho = 0.33, p = 0.04) and negatively correlated with LVEF (r = -0.416, p = 0.009). Periostin was approximately co-localized with fibrosis. Mean periostin expression was lower in patients with mild grade cardiomyocyte hypertrophy compared to those with moderate grade (p = 0.049) and lower in patients with mild grade replacement fibrosis compared to moderate grade (p = 0.036). In conclusion, periostin is overexpressed in advanced HCM, correlated with fibrosis and possibly related to cardiomyocyte hypertrophy.

## Introduction

Hypertrophic Cardiomyopathy (HCM) is the most common cardiomyopathy in the general population with an estimated prevalence of 1:200–1:500 individuals irrespective of ethnicity, geographical region and gender [1]. HCM is a genetic disorder of the sarcomere, exhibits a diverse clinical and molecular phenotype with an intricate interplay between genetic and non-genetic factors and is characterized as a "private" disease [2, 3]. Macroscopically, it is characterized by: prominent, usually asymmetrical, cardiac muscle hypertrophy mainly localized at the basal interventricular septum (IVS) which cannot be attributed to secondary causes of altered hemodynamic load and a normal to increased left ventricular ejection fraction (LVEF) without a dilated left ventricle [2]. More specifically, left ventricular hypertrophy indicative of HCM is defined by an end-diastolic ventricular septal thickness >15mm (the normal IVS thickness is 0.6–0.9cm in women and 0.6–1cm in men) [4, 5].

The hallmark characteristics of HCM regarding its clinical presentation, ranging from asymptomatic carriers to Sudden Cardiac Death, and gross anatomical features have been described as early as 1868 by the French pathologist Vulpian and in 1869 by the French pathologists Hallopeau and Liouville [6–8]. The first thorough description of its histopathological and cellular attributes was published in 1891 by Ludolf von Krehl who observed hypertrophic myocytes with large hyperchromatic nuclei, a marked increase of connective tissue in the interstitial space, perivascular fibrosis, cytoplasmic vacuolization and small vessel disease in nine autopsy cases without valvulopathy or atherosclerotic heart disease thus classifying the marked hypertrophy of the IVS as a primary heart muscle disorder [9]. In 1958, Teare provided the modern microscopic description of the classic histopathological characteristics of HCM, more specifically myocyte hypertrophy, myofibrillar disarray and interstitial fibrosis [10].

HCM is inherited in an autosomal dominant pattern and its genetic cause, for the majority of cases, are mutations in 11 sarcomeric genes with MYH7 (encoding *β*-myosin heavy chain) and MYBPC3 (encoding myosin-binding protein C) accounting for about 50% of familial cases [11]. Regarding the pathogenetic mechanism of HCM, five stages can be distinguished: the primary defect i.e. the causal mutation, initial defects such as altered mRNA transcription and calcium sensitivity stemming from each sarcomeric mutation, secondary molecular changes such as signaling pathway alterations, tertiary histological phenotypes such as myocyte hypertrophy and remodeling of the interstitium and finally quaternary clinical phenotypes ranging from arrhythmias to heart failure and SCD [2]. About 70% of patients exhibit left ventricular outflow tract obstruction (LVOTO) at rest or on exertion [12]. Patients with severe LVOTO and disabling clinical signs of drug-refractory heart failure with compatible echocardiographic findings are candidates for septal myectomy, a surgical procedure which significantly improves quality of life by alleviating life-limiting symptoms [13].

Despite the fact that the cardiomyocyte remains at center stage in HCM, broad attention has been drawn to the role of cardiac fibroblasts and extracellular matrix (ECM) remodeling in the natural course of the disease [14, 15]. The myocardial ECM is a complex supportive mesh of matricellular proteins (glycoproteins, glycosaminoglycans and proteoglycans) more specifically thrombospondin, SPARC, tenascin, osteopontin, periostin (POSTN), CCN, collagens, elastins, fibronectin, laminin, hyaluronan, hyalectans, basement and cell membrane proteoglycans, cell surface proteoglycans and small leucine rich proteoglycans [16]. The deposition of matricellular proteins by activated myofibroblasts in pressure-overloaded hearts is a modulator of hypertrophic and profibrotic responses [17]. The ECM has been proposed as an additional culprit of self-sustaining hypertrophic stimulus apart from the effect of each pathogenic mutation [18]. Additionally, impaired ECM turnover has been detected even in the early preclinical stages of the disease [19]. In animal models of HCM, cardiac fibrosis has been found to be driven by non-myocyte cellular populations [20, 21].

The current histopathological and immunohistochemical study focuses on the ECM protein periostin (POSTN) at tissue level and its correlation with myocardial fibrosis and other hallmark histopathological characteristics of HCM. Periostin was identified in 1999 and named as such due to its initial detection in the periosteum and periodontal ligament with its expression driven by TGF-*β* [22]. Periostin is a 90-kDa TGF-*β* induced secreted protein with an EMI domain (which binds to Collagen Type I, fibronectin and Notch1), a Fas1 domain comprising of 4 repeated domains (which binds to tenascin-C and BMP-1) and a C-terminal domain [23]. It is encoded at the osteoblast-specific factor 2 gene located at human chromosome 13 [23]. Periostin has been expected to be sensitive to mechanical stress stimuli, regulates collagen fibrillogenesis and the viscoelastic properties of connective tissue by playing a crucial role in collagen cross-linking [23, 24]. It is highly expressed during cardiac development, being essential to proper cardiac valve formation, is almost silenced in healthy adult hearts and re-expressed after pathologic cardiac insults [25]. Its re-expression by myofibroblasts in heart disease drives ECM remodeling thus designating it as a reliable myofibroblast marker [25]. Pressure overload and myocardial infarction are associated with markedly increased POSTN expression in animal models and POSTN has been proposed as an emerging therapeutic target to halt or reduce subsequent cardiac fibrosis [26–29]. POSTN expression is upregulated and positively correlated with myocardial fibrosis in heart failure patients [30]. The majority of studies investigating the unique role of POSTN in cardiovascular disease were performed on animal subjects [31]. To our knowledge, the current histopathological and immunohistochemical study is the first to investigate the extent of POSTN expression and its correlation with myocardial fibrosis purely on tissue samples of HCM patients who underwent septal myectomy for the relief of drug refractory symptoms.

## Materials and methods

The present study builds on the already published, histopathological study of septal myectomy specimens from a Greek cohort [32] and shares with it the following components: study population, stored tissue blocks, observed hallmark histopathological parameters, digitization procedures and parts of the methodology as will be described in greater detail below.

### Study population

The HCM registry of the 1^st^ Cardiology Department at AHEPA University Hospital in Thessaloniki and the database of the Cardiothoracic Surgery Department at St. Luke’s Hospital in Thessaloniki were retrospectively reviewed. All myectomes were performed by the same, highly specialized cardiac surgery team at a single Cardiothoracic Surgery Department in Thessaloniki. Patients were considered to have a diagnosis of primary HCM on the basis of clinical criteria. Patients with left ventricular hypertrophy related to aortic valve stenosis or hypertension were excluded. A total of thirty nine (39) patients who underwent septal myectomy for the relief of drug refractory symptoms from 2007 to 2017 were identified. The control group consisted of five (5) individuals without known cardiovascular disease who died from non-cardiac causes as documented by postmortem autopsy and their paraffin-embedded tissue blocks containing cardiac tissue from the IVS were available for analysis. The current study was conducted in 2022. Informed consent regarding the participation in the study and the processing of anonymized data and tissue samples was provided by all patients. The authors had access to information that could identify individual participants during the stage of data collection. After completion of collection, all data were anonymized by assigning a single numerical identifier to each participant. The study was conducted in accordance with the Declaration of Helsinki, and approved by the Ethics Committee of the Aristotle University of Thessaloniki Department of Medicine (approval number 3654, 2 May 2018).

### Demographic and clinical characteristics

Patients’ age, gender, diagnosis and NYHA functional class (I to IV) were obtained from their medical records. Preoperative echocardiographic parameters were also obtained from which Left Ventricular Ejection Fraction (LVEF), maximal IVS thickness and peak resting LVOT pressure gradient were included in the analysis.

### Surgical technique for septal myectomy

The surgical technique applied for septal myectomy at was the one introduced by Andrew Morrow [3, 33]. A brief description of the technique is provided in section "Surgical technique for septal myectomy" of S1 Appendix.

### Histopathology and immunohistochemistry

#### Routine histopathology

Tissue samples from each myectomy procedure were processed for histopathological examination and two stains were prepared, hematoxylin and eosin (H+E) and Masson’s Trichrome, as described in previous work and as explained in the section "Histopathology" of S1 Appendix [32]. Masson’s Trichrome stain was used to qualitatively and quantitatively measure fibrosis. Slides were evaluated with light microscopy as well as with digital means by two pathologists experienced in cardiovascular pathology (the authors N.S.I. and S.M.). Hallmark histopathological characteristics and their qualitative or semi-quantitative grading (absent—mild—moderate—severe, thoroughly described in the section "Histopathology" of S1 Appendix) were obtained from previous work [32] and are listed as follows: myocyte hypertrophy, cytoplasmic vacuolization, subendocardial fibrosis, interstitial fibrosis, replacement fibrosis, myocardial disarray and microvascular stenosis.

#### Immunohistochemistry

A third consecutive section of 4μm (two sections already obtained for H+E and Masson’s Trichrome as described in the previous sub-subsection) was cut from each myectomy paraffin-embedded tissue block using a microtome and another section of 4μm from each tissue block of the control group. Sections were placed on positively charged microscope slides (Süssefrost Plus, Süsse Labortechnik, Gudensberg, Germany). Immunostaining was performed on the BOND-MAX fully automated IHC system (Leica Biosystems Melbourne Pty Ltd, Victoria, Australia). Crucial steps of the automated immunostaining protocol are described as follows. Tissues were initially deparaffinized and Heat Induced Epitope Retrieval (HIER) was performed by pretreating tissues with a citrate based pH 6 epitope retrieval solution (AR9961 Bond TM Epitope Retrieval 1, Leica Biosystems Newcastle Ltd, Newcastle upon Tyne, United Kingdom) at 100 *◦*C for 20 minutes. After peroxide blocking and washing steps, according to the BOND-MAX automated protocol, tissues were incubated with POSTN unconjugated monoclonal antibody (1:100, sc-398631, Santa Cruz Biotechnology, Dallas, Texas, USA). HRP-3,3-Diaminobenzidine (DAB) method was used for the chromogenic detection of immunostaining.

### Digital pathology and quantification of total fibrosis and periostin staining

All slides stained with Masson’s Trichrome and POSTN were digitized in Tag Image File (.TIF) format with a whole slide scanner using a scan magnification of 40X (VENTANA DP 200 slide scanner, Roche Diagnostics, Basel, Switzerland). Datasets containing the digital slides of each of the above-mentioned stains were created and imported to QuPath version 0.4.1 for viewing and analysis [34]. An initial thresholder to detect and segment tissue on the digital slides was created and applied on both datasets. One thresholder was created for the Masson’s Trichrome dataset to detect and quantify the total percentage of fibrosis (stained blue) as the fraction of the total tissue area for each slide. Another thresholder was created to detect and quantify the total percentage of POSTN immunostaining (stained brown) as the fraction of the total tissue area for each slide. Quantification results were verified by two pathologists experienced in cardiovascular pathology (the authors N.S.I. and S.M.) to detect under- or overestimation of percentages due to staining inhomogeneities of certain slides. In such cases the thresholders were tailored to each affected slide by adjusting thresholder resolution, threshold values and smoothing sigma value aiming to achieve an accurate detection. Details regarding the creation and application of each thresholder are presented in section "Digital pathology" of S1 Appendix.

### Statistical analysis

Continuous data were tested for normality by using the Shapiro-Wilk test which is more powerful for small sample sizes [35, 36]. Normally distributed data are expressed as "mean *±* standard deviation" whereas non-normally distributed as "median(interquartile range)". The Welch’s t-test was used to compare the means of continuous variables between two groups exhibiting unequal variances. One-way ANOVA with Holm-Šídák post-hoc test was used to compare the means of continuous variables between three or more groups. Equal variances of continuous data between groups were tested and confirmed by using the Brown-Forsythe test and Bartlett’s test before the use of one-way ANOVA. Multiplicity adjusted p-values were calculated for one-way ANOVA. In case of ANOVA assumptions violation, the non-parametric Kruskal-Wallis test with Dunn’s multiple comparisons post-hoc test was used. Pearson’s r was used to test for correlations between continuous normally distributed variables. Spearman’s Rho was used to test for correlations between continuous variables violating the assumptions of Pearson’s r. All statistical tests were two-tailed and considered significant for p≤0.05. Statistical analysis was performed using SPPS Software (version 25, IBM, SPSS Statistics, Chicago, Illinois USA) and GraphPad Prism (version 9, GraphPad Software, San Diego, California USA).

## Results

### Patient characteristics

The study included thirty-nine (39) patients, 22 males (56.4%) and 17 females. Mean age at myectomy was 53.9 *±* 16.7 years, ranging from 12 to 79 years. Patients presented with a median NYHA functional class value of 3 (Class III). Mean LVEF was (70.1 *±* 8.93) %. The median value for maximal IVS thickness was 2.08(0.34)cm. Mean value of peak resting LVOT pressure gradient (LVOT PG) as estimated by echocardiography was (108.11 *±* 44.2)mmHg. The control group comprised 3 males and 2 females with a mean age of 50.2 *±* 4.68 years. Patient characteristics are summarized in Table 1.

**Table 1:** Patient Characteristics. Study population N = 39.

**Age in years**	Mean ± SD	53.9 ± 16.7
	Range	12–79
**Gender**	Male (% of N)	22 (56.4)
	Female (% of N)	17 (43.6)
**NYHA Functional Class**	NYHA I and II (% of N)	28.2
	NYHA III and IV (% of N)	71.8
**LVEF%**	Mean ± SD	70.1 ± 8.93
**Maximal IVS thickness (cm)**	Median(IQR)	2.08(0.34)
**Peak resting** **LVOT PG (mmHg)**	Mean ± SD	108.11 ± 44.2

### Histopathological features

Histopathological features of the study population were presented in previous work [30]. In brief, all patients exhibited a certain grade of myocyte hypertrophy with the majority (64.1%) exhibiting moderate grade hypertrophy. Regarding the rest of the hallmark histopathological characteristics of HCM which were observed, the median grades for each one were as follows: mild cytoplasmic vacuolization, moderate subendocardial fibrosis, moderate interstitial fibrosis, mild replacement fibrosis, moderate myocardial disarray and mild microvascular stenosis. Histopathological features are summarized in Table 2.

**Table 2:** Histopathological features of the study population (N = 39).

Feature	Grade	Number of patients (% of N)
Myocyte hypertrophy	Mild	8 (20.5)
	Moderate	25 (64.1)
	Severe	6 (15.4)
Cytoplasmic vacuolization	Absent	3 (7.7)
	Mild	23 (59)
	Moderate	10 (25.6)
	Severe	3 (7.7)
Subendocardial fibrosis	Mild	12 (30.8)
	Moderate	21 (53.8)
	Severe	6 (15.4)
Interstitial fibrosis	Mild	2 (5.1)
	Moderate	22 (56.4)
	Severe	15 (38.5)
Replacement fibrosis	Mild	29 (74.4)
	Moderate	7 (17.9)
	Severe	3 (7.7)
Myocardial disarray	Mild	16 (41)
	Moderate	18 (46.2)
	Severe	5 (12.8)
Microvascular stenosis	Mild	25 (64.1)
	Moderate	13 (33.3)
	Severe	1 (2.6)

### Periostin expression in HCM versus controls

**Fig 1:**
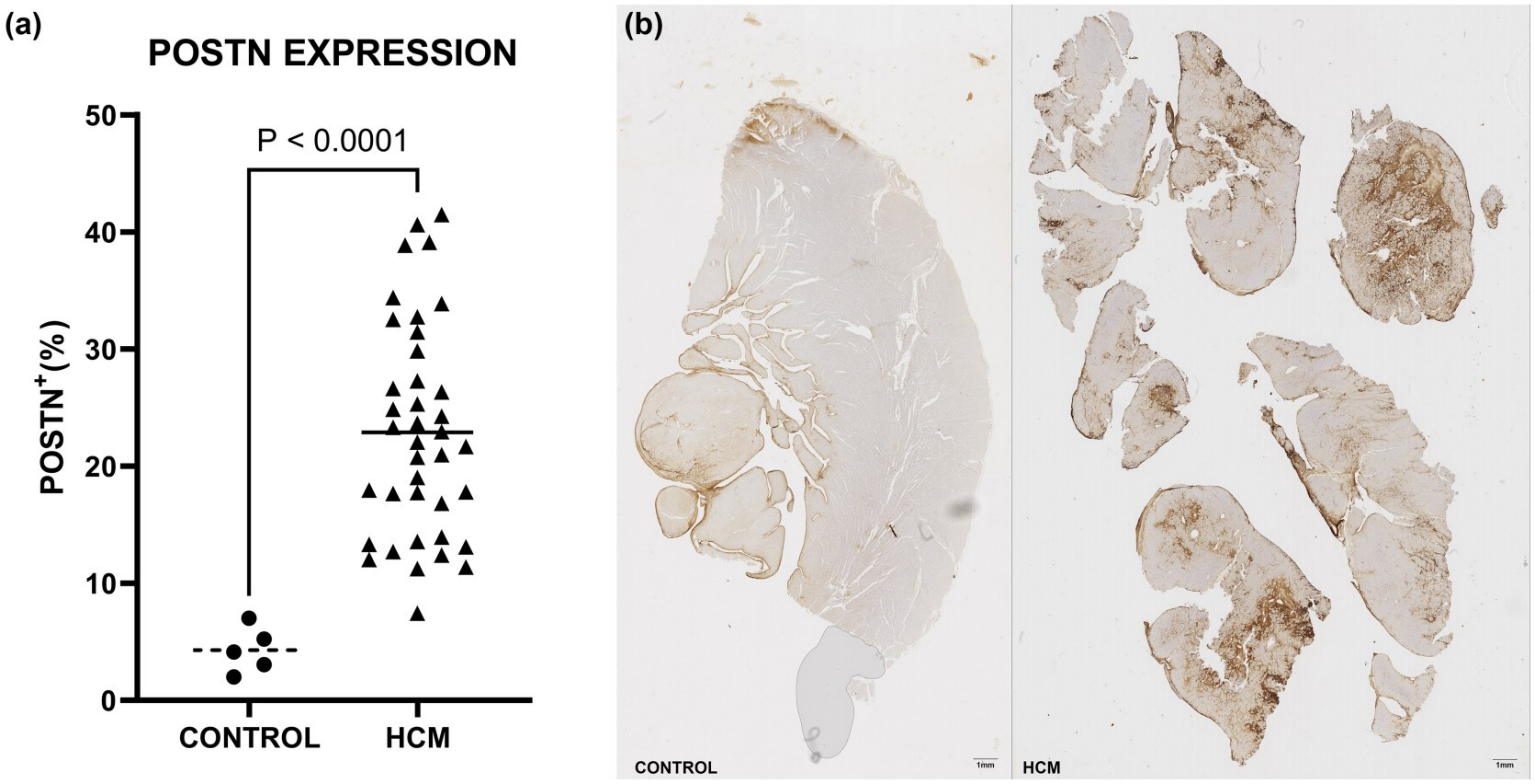
(a) Scatter dot plot of Periostin expression in HCM versus Control myocardial tissue. The mean of the Control group is marked with an interrupted line whereas the mean of the HCM group with a continuous line. (b) Side by side whole slide comparison of POSTN immunostaining extent in control tissue (left, 4.15% POSTN+) and HCM tissue (right, 24.84% POSTN+).

The mean percentage of POSTN^+^ immunostaining in HCM patients was (22.9 ± 9.11)%, significantly higher [t(31.83) = 10.96, p<0.0001] than in control hearts which exhibited a mean of (4.29 ± 1.94)% as graphed in Fig 1.

### Periostin correlation with fibrosis

**Fig 2:**
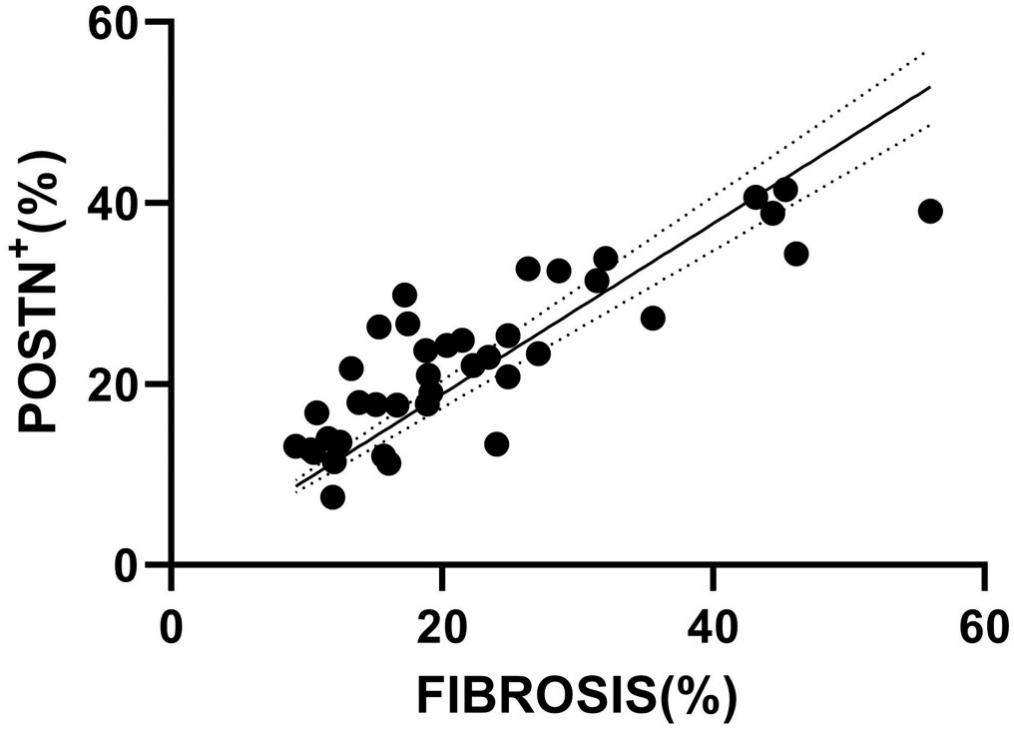
Correlation plot with fitted line visualizing the positive correlation between the extent of POSTN expression and fibrosis. Spearman’s Rho = 0.82, p<0.001.

Periostin was found to be dispersedly expressed in HCM myocardial tissue following approximately the same distribution as fibrosis (co-localization is analyzed in sub subsection 3.4.1.). Mean extent of POSTN^+^ staining was (22.9 ± 9.11)% ranging from 7.45 to 41.46%. The median of fibrosis extent was 18.99(17.92)% ranging from 9.19 to 56.05%. There was a strong positive correlation (r = 0.82, p<0.001) between the extent of POSTN expression and the extent of fibrosis (Fig 2).

### Periostin co-localization with fibrosis

**Fig 3:**
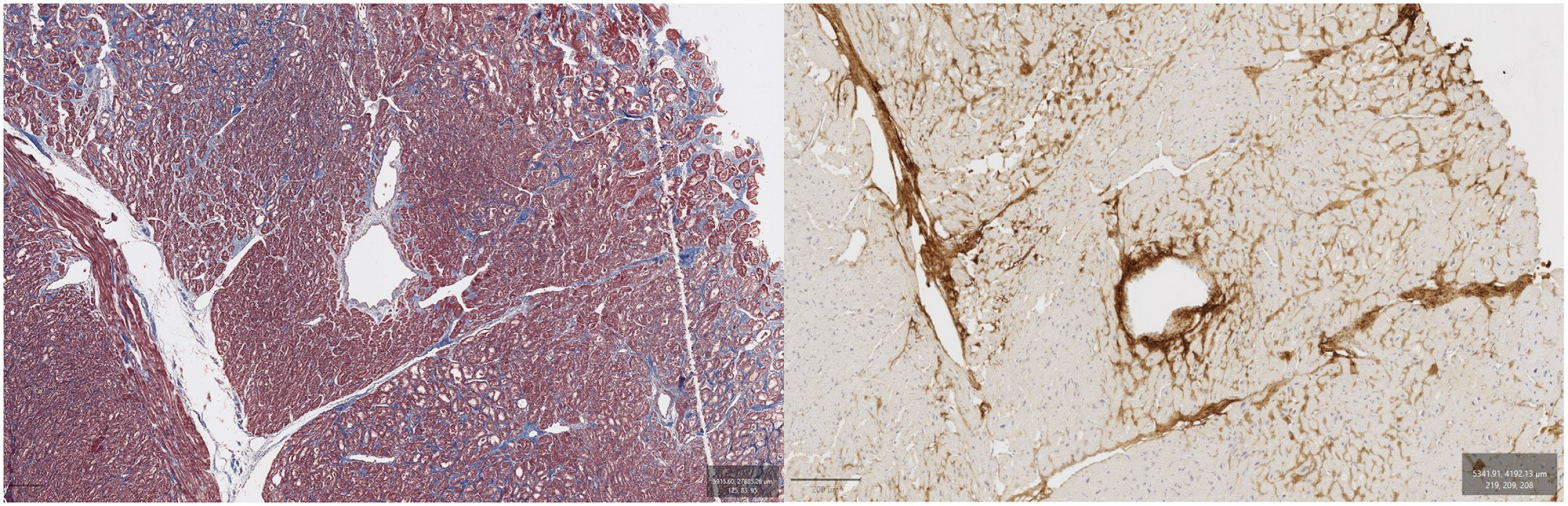
Female patient, 12 years old. Masson’s Trichrome (left) and POSTN immunostaining (DAB, right). The central vessel can be used as an orientation point.

**Fig 4:**
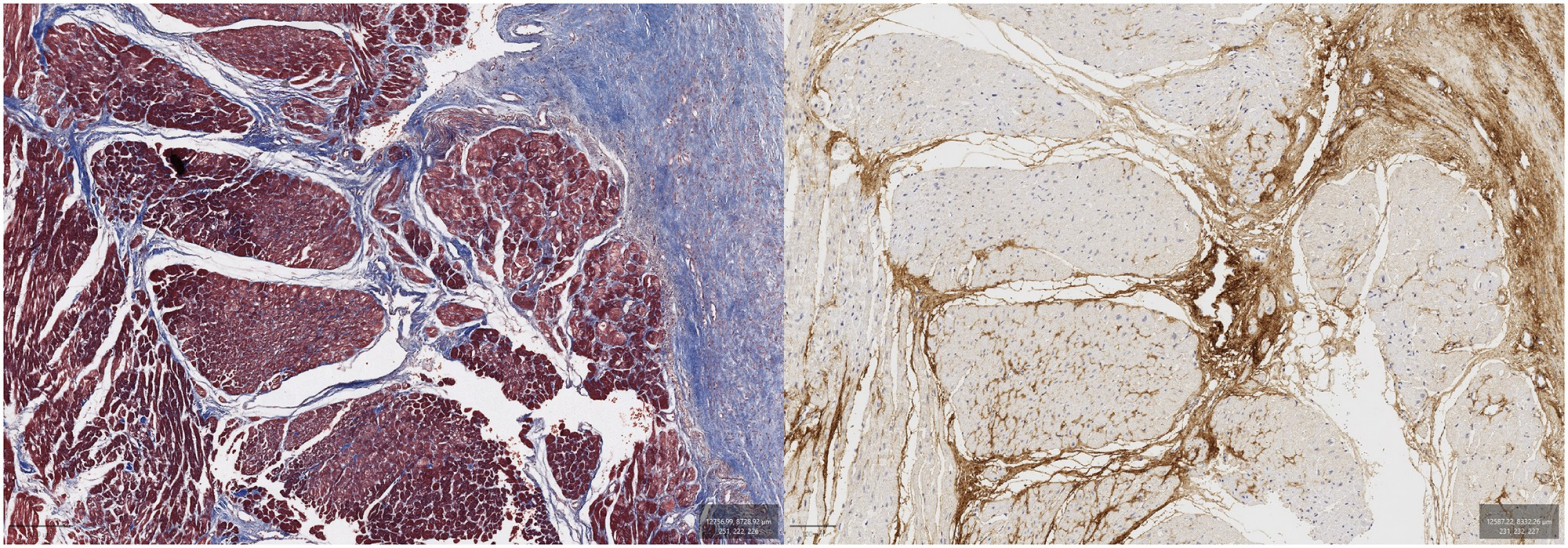
Female patient, 61 years old. Masson’s Trichrome (left) and POSTN immunostaining (DAB, right).

**Fig 5:**
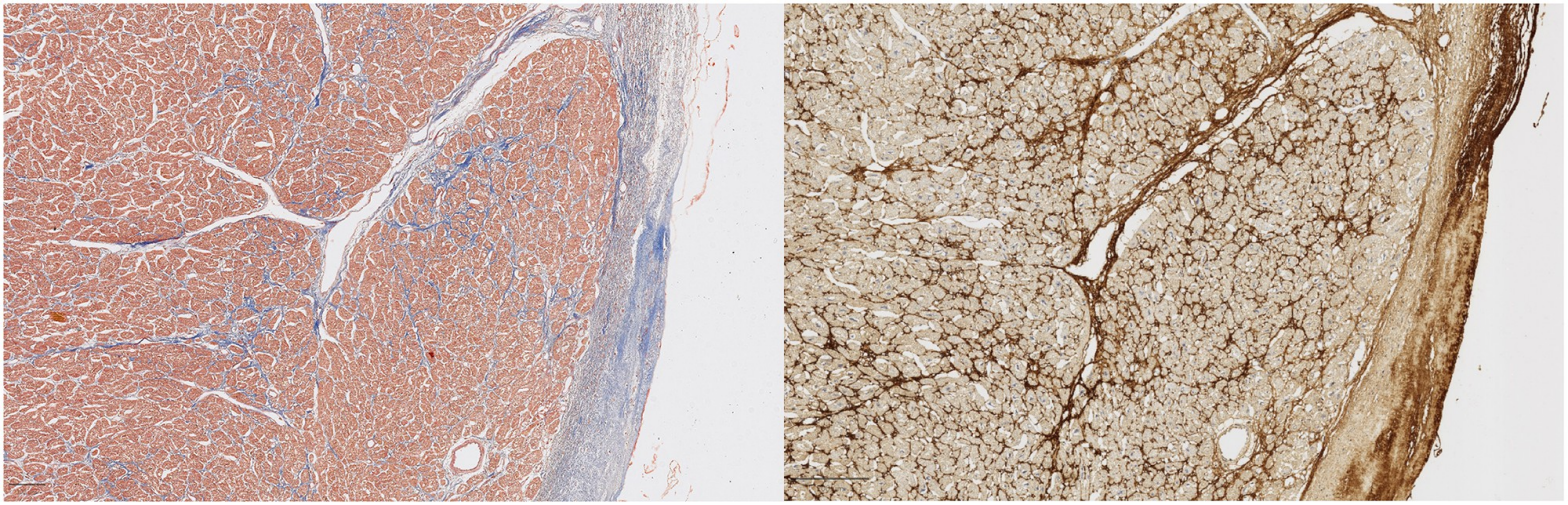
Female patient, 63 years old. Masson’s Trichrome (left) and POSTN immunostaining (DAB, right). The vessel located at the bottom right of each image can be used as an orientation point.

Examination of consecutive sections stained with Masson’s Trichrome and POSTN revealed that POSTN was approximately co-localized with fibrosis. Co-localization was highly evident in areas of replacement fibrosis as well as perivascularly (Figs 3 to 5).

### Periostin expression in relation to histopathological features

**Fig 6:**
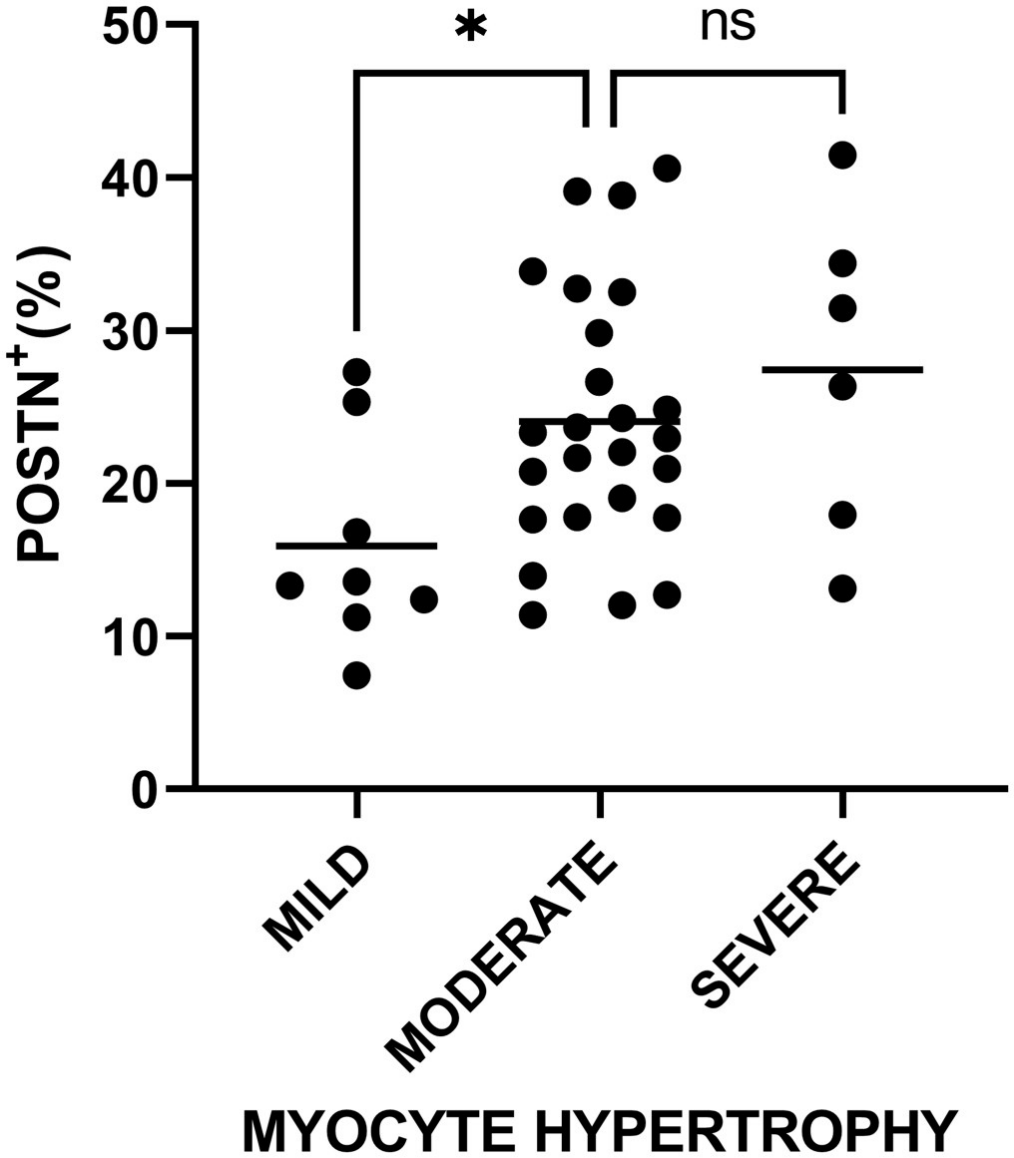
Scatter dot plot of Periostin expression among grades of myocyte hypertrophy. The mean value for each grade is marked with a continuous line. *p = 0.049, ns = not significant.

A one-way ANOVA revealed that there was a statistically significant difference in mean POSTN^+^ immunostaining between at least two grades of myocyte hypertrophy (F(2,36) = 3.76, p = 0.033). Holm-Šídák post-hoc test concluded that the mean value of POSTN^+^ immunostaining was significantly different between mild (mean = 15.93%) and moderate (mean = 24.04%) myocyte hypertrophy (p = 0.049, difference of means = -8.11) and between mild and severe (mean = 27.45%) myocyte hypertrophy (p = 0.049, difference of means = -11.52) as shown in Fig 6. Interestingly, no significant difference in median values of fibrosis extent among grades of myocyte hypertrophy was detected (see section "Fibrosis extent among grades of myocyte hypertrophy" of S1 Appendix).

**Fig 7:**
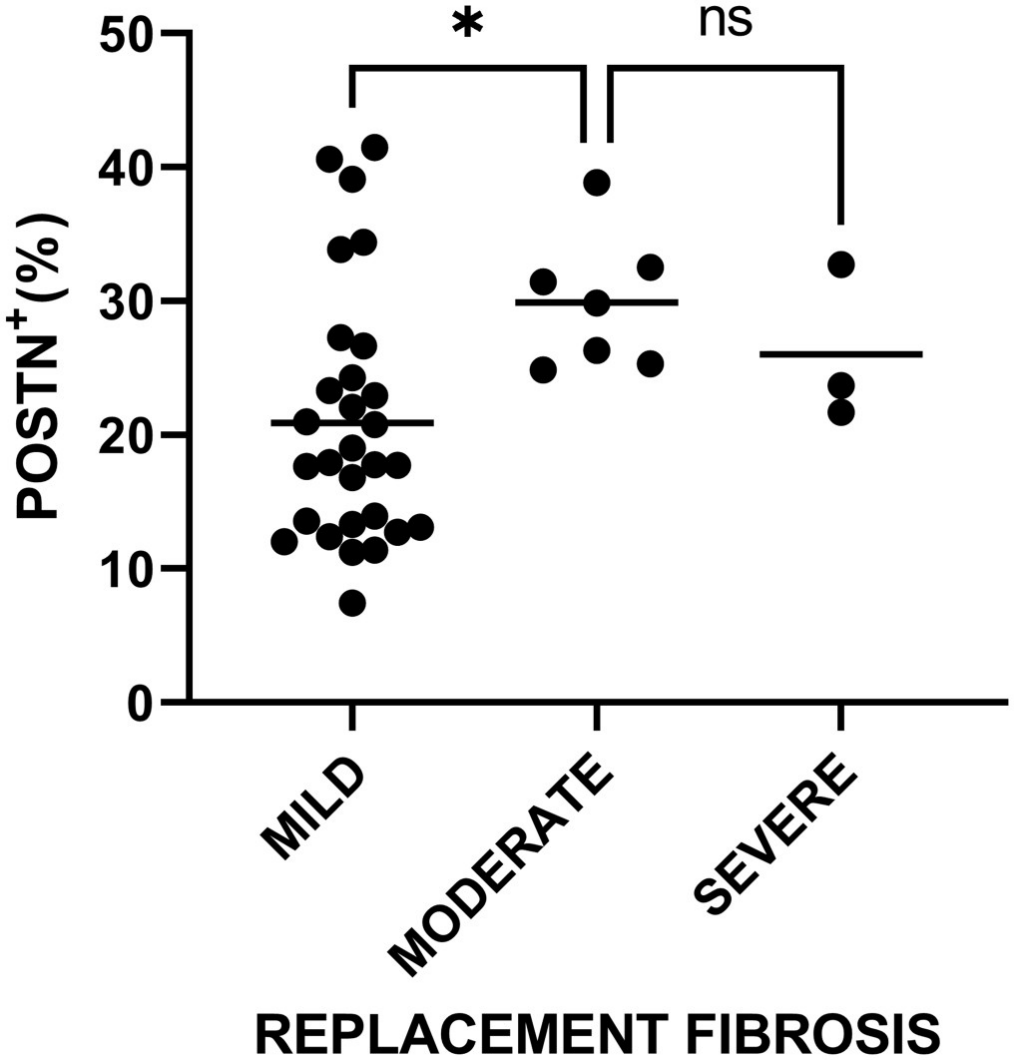
Scatter dot plot of Periostin expression among grades of replacement fibrosis. The mean value for each grade is marked with a continuous line. * p = 0.036, ns = not significant.

A one-way ANOVA revealed that there was a statistically significant difference in mean POSTN^+^ immunostaining between at least two grades of replacement fibrosis (F(2,36) = 3.285, p = 0.049). Holm-Šídák post-hoc test concluded that the mean value of POSTN^+^ immunostaining was significantly different between mild (mean = 20.89%) and moderate (mean = 29.88%) replacement fibrosis (p = 0.036, difference of means = -8.99) as shown in Fig 7. No significant difference in mean POSTN expression was found between groups of the rest histopathological features.

### Periostin correlation with patient characteristics (demographics and echocardiographic parameters)

**Fig 8:**
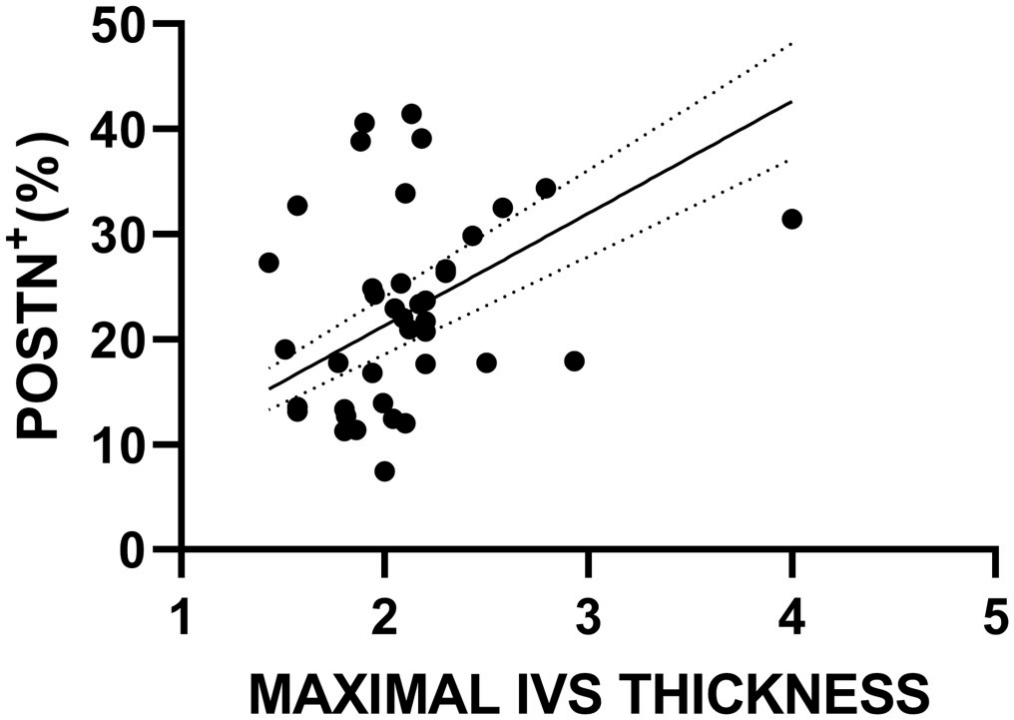
Correlation plot with fitted line visualizing the positive correlation between the extent of POSTN expression and maximal IVS thickness (Spearman’s Rho = 0.33, p = 0.04).

**Fig 9:**
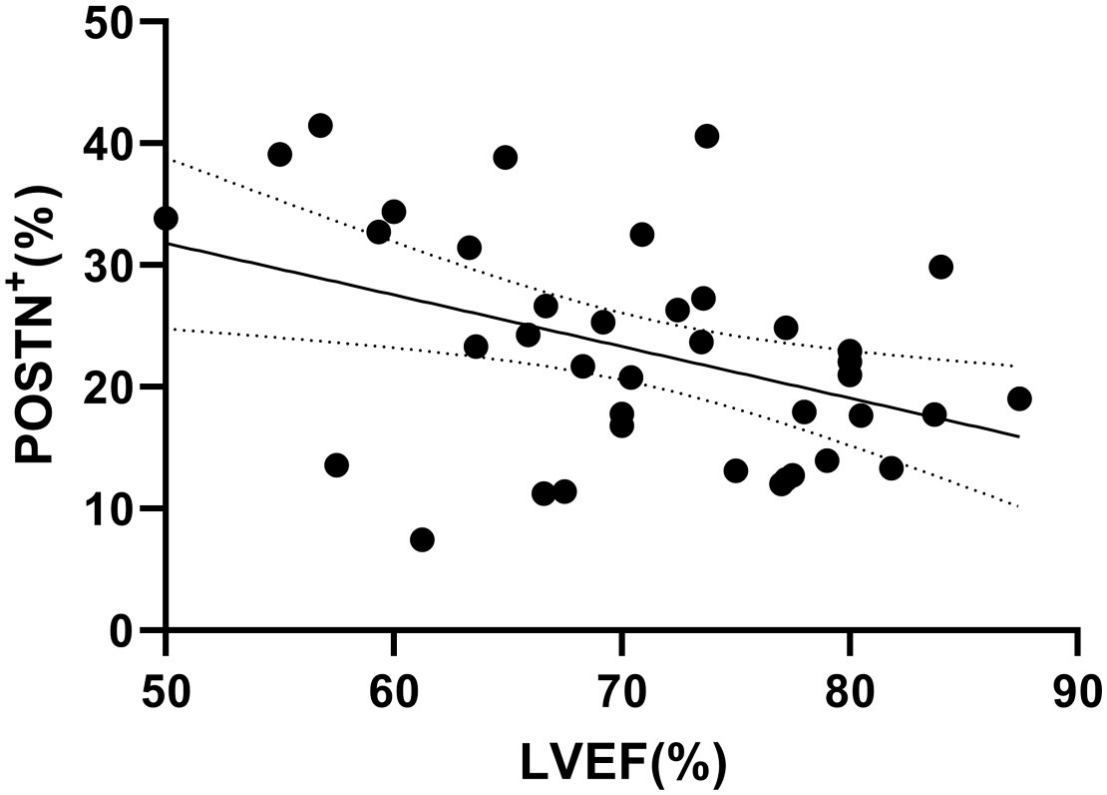
Correlation plot with fitted line visualizing the negative correlation between the extent of POSTN expression and LVEF (Pearson’s r = -0.416, p = 0.009).

The extent of POSTN^+^ immunostaining was positively correlated with maximal IVS thickness (r = 0.33, p = 0.04) and negatively correlated with LVEF (r = -0.416, p = 0.009) (Figs 8 and 9 respectively). The extent of POSTN expression was not correlated with age, gender, NYHA functional class and peak LVOT PG.

## Discussion

The current study highlights the overexpression of the matricellular protein Periostin in cardiac tissue of HCM patients who underwent septal myectomy as compared to normal hearts. Hence, the study refers purely to a population of HCM patients at an advanced stage of the disease. It also shows that the extent of POSTN expression is correlated with that of fibrosis. In comparison, the study of Zhao et al. which reached similar results as ours, predominantly included dilated cardiomyopathy patients and two HCM patients, all diagnosed with advanced heart failure [30]. Our study reveals a fair but significant correlation of the extent of POSTN expression with maximal IVS thickness. Moreover, the mean extent of POSTN expression differs significantly between mild and moderate myocyte hypertrophy as well as mild and moderate replacement fibrosis, with mild grades of both features exhibiting lower mean extent of POSTN expression. Mean extent of POSTN expression does not differ between moderate and severe myocyte hypertrophy and replacement fibrosis. Our findings are consistent with the current pathophysiological model of cardiomyocyte—fibroblast interaction according to which ongoing LV pressure overload stimulates the initial differentiation of mature fibroblasts to POSTN-expressing activated fibroblasts and ultimately to myofibroblasts which maintain scar formation (replacement fibrosis) [25]. Differentiation of resident cardiac fibroblasts to POSTN-expressing myofibroblasts is known to be driven by TGFβ signalling which is activated in response to chemical and mechanical stressors [20, 37]. Secreted POSTN is also considered a stimulus for the differentiation of activated fibroblasts to myofibroblasts [25]. It is worth adding that cardiac fibroblasts have been found to induce cardiomyocyte hypertrophy via specific paracrine miRNA signaling and that pathologically remodeled ECM can impair contractility when co-existent with healthy cardiomyocytes [18, 38]. POSTN itself has been implicated in inducing cardiac hypertrophy in animal and human studies [29, 39]. These interactive mechanisms support the concept of a self sustaining pathophysiology at advanced stages of HCM, a concept which is further reinforced after the observation that the drug Mavacamten could not halt disease progression after the onset of hypertrophy, thus raising the hypothesis that the ECM plays a crucial role in stimulating hypertrophy even in the preclinical stages [38]. Building on the beforementioned mechanisms we could inferentially suggest that the findings of our study support a bidirectional interplay between POSTN and cardiomyocytes which could spark further research: progression of myocyte hypertrophy from mild to moderate grade is associated with a significant increase in mean POSTN expression, an increase which remains approximately the same in severe grade of hypertrophy. The same pattern applies to mean POSTN expression among grades of replacement fibrosis. The "stabilization" of mean POSTN expression between moderate and severe myocyte hypertrophy and replacement fibrosis indicates a "point of no return" regarding the intricate process of ECM remodeling. Apparently, that specific point is designated before the onset of moderate cardiomyocyte hypertrophy raising the question whether POSTN inhibition at the earliest possible stage of the disease could halt its progression. Such experimental approach has been employed in the case of renal fibrosis where POSTN knockout in mice as well as POSTN blockade in mice normally expressing POSTN were both correlated with reduced levels of profibrotic markers after mechanical stress in vivo and in vitro [40]. In the revolutionizing era of Mavacamten which targets cardiomyocyte mechanics, a concomitant therapeutic regimen targeting ECM remodeling would be of paramount importance and periostin could serve as an appealing target. It should be noted that our study poses certain limitations. It is retrospective in nature with a small sample size thus it is prone to selection bias of controls and documents correlation, not causation. It only includes patients at an advanced stage of the disease. The control group is small but was deemed adequate considering the fact that apparently healthy myocardial tissue is hard to obtain and that periostin is already known to be minimally expressed in healthy adult myocardium. As a proposal and framework for further research, additional stains could be performed such as: a) sirius red staining to obtain the quality and maturation of the deposited collagen and possibly seek a correlation between loosely and dense packed collagen with periostin expression, b) co-staining with other markers of activated fibroblasts and myofibroblasts e.g. a-SMA and co-staining with proliferation markers. Our team currently studies the expression of fibroblast marker Transcription factor 21 (Tcf21) and its possible correlation with fibrosis. Further research could also be directed to the role of Long noncoding RNAs (lncRNAs) in the regulation of cardiac profibrotic response. The lncRNAs Wisper, Meg3, Safe and Cfast have been identified as potent regulators of cardiac fibrosis and their depletion has been linked to POSTN downregulation [41].

## Supporting information

S1 Appendix Study methods in detail and secondary result. S1 Appendix file contains detailed information regarding the surgical technique for septal myectomy, histopathology, digital pathology and fibrosis extent among grades of myocyte hypertrophy as stated in the main text. The minimal dataset underlying the results is available as a Microsoft Excel spreadsheet file (S1 Dataset.xlsx). Moreover, the following supporting information can be downloaded at: https://drive.google.com/drive/folders/1DB6UqdoonyNovjC-1XUPAxaFbaiI VP92?usp=sharing, thresholder script files (.json) which were used in QuPath to detect tissue and quantify fibrosis and periostin, each named as described in section "Digital pathology" of S1 Appendix.
(PDF)

S1 Dataset Dataset underlying the results of the study. (XLSX)
