# Supplementary material for: Periostin is overexpressed, correlated with fibrosis and differs among grades of cardiomyocyte hypertrophy in myectomy tissue of patients with hypertrophic cardiomyopathy
Source: PLoS One. 2023 Nov 8;18(11):e0293427. doi: 10.1371/journal.pone.0293427 (PMC10631645; doi:10.1371/journal.pone.0293427)
Supplement: S1 Appendix — file contains detailed information regarding the surgical technique for septal myectomy, histopathology, digital pathology and fibrosis extent among grades of myocyte hypertrophy as stated in the main text. The minimal dataset underlying the results is available as a Microsoft Excel spreadsheet file (S1 Dataset.xlsx). Moreover, the following supporting information can be downloaded at: https://drive.google.com/drive/folders/1DB6UqdoonyNovjC-1XUPAxaFbaiI VP92?usp=sharing, thresholder script files (.json) which were used in QuPath to detect tissue and quantify fibrosis and periostin, each named as described in section "Digital pathology" of S1 Appendix. (PDF) [file pone.0293427.s001.pdf]

S1 APPENDIX

Surgical technique for septal myectomy

After sternotomy, a vertical aortotomy is performed thus initiating the technique. The basal IVS is visualized through the aortotomy and is located below the right coronary cusp of the aortic valve. Two parallel longitudinal incisions are performed in the basal IVS. A third vertical incision below the right coronary cusp of the aortic valve is performed and connects the two parallel longitudinal incisions. The beforementioned incisions form a trough of approximately 4-5cm in length. This trough extends to just beyond the mitral-septal contact. When required, the incision was extended distally to the base of the papillary muscles. The procedure aimed to resect 5-10 grams of tissue. Transesophageal echocardiography was used throughout the procedure to monitor and confirm the extent of myectomy.

Histopathology

Tissue from each septal myectomy procedure was placed in 10% Neutral Buffered Formalin (NBF) immediately after excision. After NBF fixation tissue underwent dehydration over ascending alcohols (ethanol), was cleared in xylene and subsequently embedded in paraffin. Paraffin-embedded blocks contained tissue covering the whole thickness of the surgical specimen from the endocardial to the myocardial layer. Blocks were meticulously stored under controlled conditions. Using a microtome, two 4μm thin sections were cut from each block and stained with H+E and Masson's Trichrome each. Histopathological parameters were recorded in a pathology report form and were defined as follows:

- Myocyte Hypertrophy: the presence of a hyperchromatic and enlarged nucleus and a myocyte diameter greater than 20μm (approximately greater than the diameter of three red blood cells). Grading was semi-quantitative and hypertrophy was considered mild if overall myocyte diameters were 21-29μm (3-4 RBCs), moderate if 30-38μm (4-5 RBCs) and severe if >38μm (>5 RBCs)
- Cytoplasmic vacuolization: graded qualitatively as absent, mild, moderate and severe on the basis of the pathologists' experience
- Subendocardial fibrosis: graded qualitatively as absent, mild, moderate and severe on the basis of the pathologists' experience
- Interstitial fibrosis: graded semi-quantitatively from Masson's staining as absent, mild if its extent was $\leq$ 30% of the myocardial area, moderate if >30 and <60% and

severe if ≥60%

• Replacement fibrosis: graded semi-quantitatively from Masson’s staining as absent, mild if its extent was ≤ 30% of the myocardial area, moderate if >30 and <60% and severe if ≥60%

• Myocardial disarray: defined as the presence of a bizarre disorganization of myocardial fibers sometimes following whirling, herringbone or other patterns. It was graded semi-quantitatively as absent, mild if its extent was 1-25% of the myocardial area, moderate if 26-50% and severe if >50%

• Microvascular stenosis: graded semi-quantitatively as absent, mild if luminal stenosis was <30%, moderate if ≥30 and <60% and severe if ≥60%.

Digital pathology

Before analysis of each dataset (Masson’s Trichrome and POSTN) on QuPath version 0.4.1. stain vectors were set according to the relevant QuPath tutorial available at https://qupath.readthedocs.io/en/stable/docs/tutorials/separating_stains.html .

The initial thresholders created to detect tissue were named "TISSUE DETECTOR MASSON’S" and "TISSUE DETECTOR POSTN". After detection a unified object exclusively containing tissue was created (Figure A1). The files (.json) for each of the before-mentioned thresholders are available for download at the Supplementary Materials section. They were created according to guidelines at the QuPath tutorials website and are available at https://qupath.readthedocs.io/en/stable/docs/tutorials/thresholding.html.

The thresholders created to quantify fibrosis and POSTN immunostaining were named "FIBROSIS DETECTOR" (Figure A2) and "PERIOSTIN DETECTOR" (Figure A3) respectively and the files for each one (.json) are available for download at the Supplementary Materials section. They were created according to guidelines provided at the QuPath tutorials website which are available at https://qupath.readthedocs.io/en/stable/do cs/tutorials/measuring_areas.html .

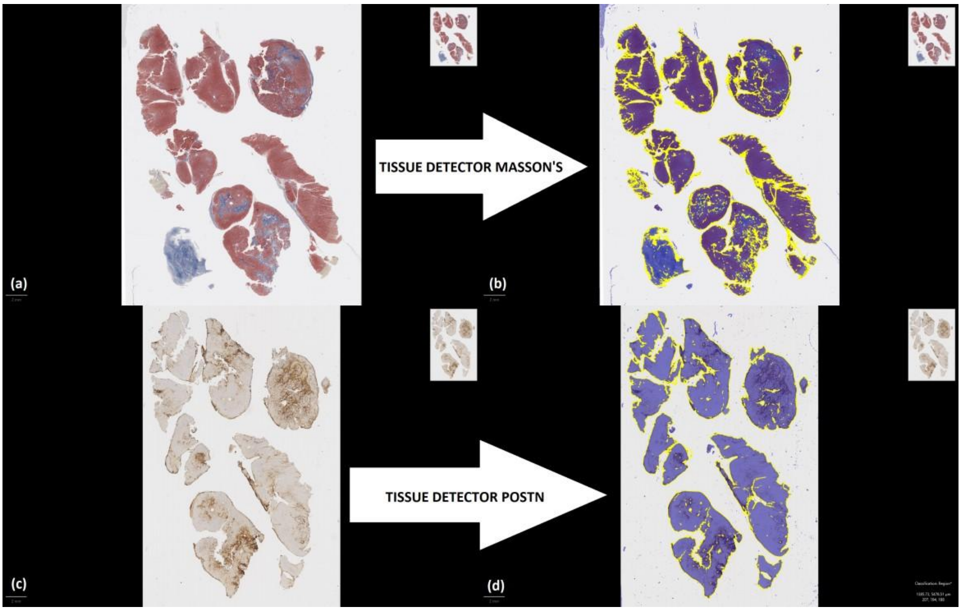

Figure A1: Tissue detection by using the initial thresholders "TISSUE DETECTOR MASSON’S" and "TISSUE DETECTOR POSTN". Both Masson’s and POSTN digital slides are consecutive tissue sections which belong to the same case. (a) Masson’s Trichrome slide before thresholding; (b) Masson’s Trichrome slide after thresholding. Detected tissue is overlaid with blue color and the unified object created is delineated with yellow; (c) POSTN immunostaining slide before thresholding; (d) POSTN immunostaining slide after thresholding. Detected tissue overlaid with blue color and the unified object created is delineated with yellow.

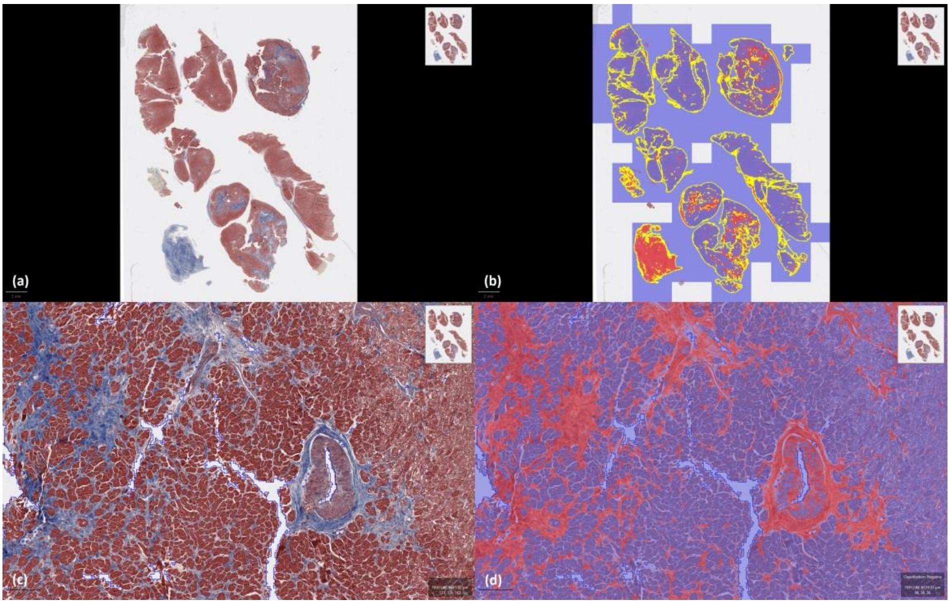

Figure A2: Fibrosis detection and quantification with the thresholder "FIBROSIS DETECTOR". (a) Masson's Trichrome digital slide before detection; (b) Detected fibrosis is overlaid with red color and considered by QuPath as positive area whereas areas overlaid with blue are considered negative areas. Fibrosis percentage is calculated as the fraction of the total tissue area delineated with yellow; (c) Zoomed view from the same specimen before fibrosis detection; (d) The same zoomed view after fibrosis detection with positive areas overlaid with red color.

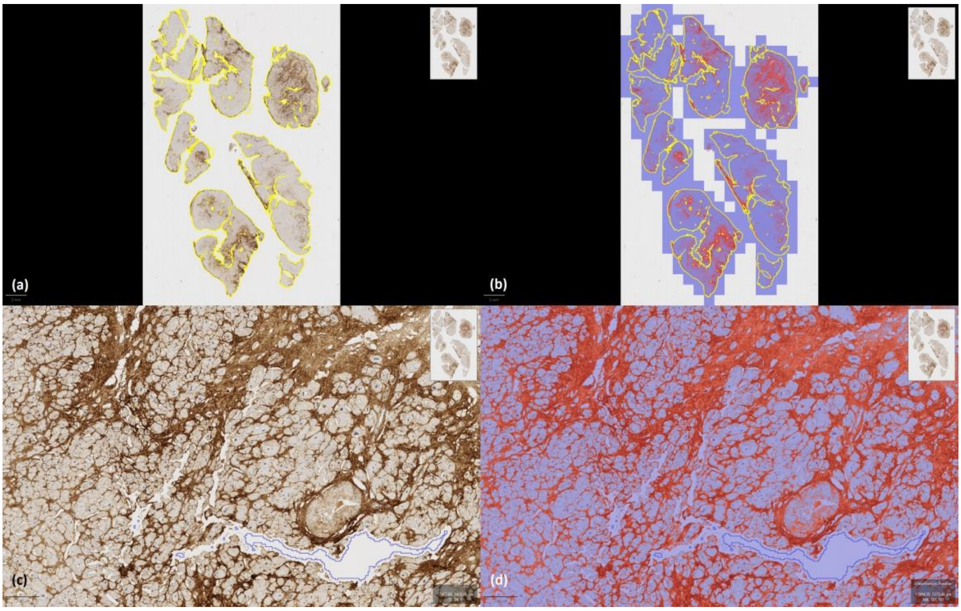

Figure A3: POSTN immunostaining detection and quantification with the thresholder "POSTN DETECTOR". (a) POSTN immunostaining digital slide before detection; (b) Detected POSTN is overlaid with red color and considered by QuPath as positive area whereas areas overlaid with blue are considered negative areas. POSTN immunostaining percentage is calculated as the fraction of the total tissue area delineated with yellow; (c) Zoomed view from the same specimen before POSTN detection; (d) The same zoomed view after POSTN detection with positive areas overlaid with red color.

Fibrosis extent among grades of myocyte hypertrophy

A Kruskal-Wallis test detected no significant difference in median values of fibrosis extent among grades of myocyte hypertrophy [H(2) = 1.758, p = 0.415] (Figure A4).

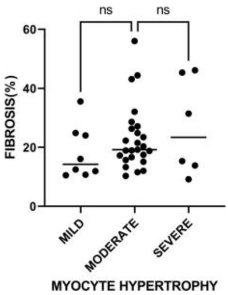

Figure A4: Scatter dot plot of fibrosis extent (%) among grades of myocyte hypertrophy. The median value for each grade is marked with a continuous line. ns = not significant.
